# Examining factor structure and psychometric properties of Ethical Leadership Questionnaire with Healthcare Professionals in Saudi Arabia

**DOI:** 10.12669/pjms.41.1.10861

**Published:** 2025-01

**Authors:** Badr K. Aldhmadi, Rakesh Kumar, Bilesha Perera, Mohammad A. Algarni

**Affiliations:** 1 Badr K. Aldhmadi, Ph.D Department of Health Management, College of Public Health and Health Informatics, University of Ha’il, Ha’il, Saudi Arabia; 2 Rakesh Kumar, Ph.D Department of Health Management, College of Public Health and Health Informatics, University of Ha’il, Ha’il, Saudi Arabia; 3 Bilesha Perera, MSc., Ph.D Faculty of Medicine, University of Ruhuna, Galle, Sri Lanka. Department of Health Management, College of Public Health and Health Informatics, University of Ha’il, Ha’il, Saudi Arabia; 4 Mohammad A. Algarni, Ph.D Faculty of Economic and Administration, King Abdulaziz University, Jeddah, Saudi Arabia

**Keywords:** Ethical Behavior, Ethical Leadership Questionnaire, Factor structure, Psychometric properties, Healthcare professional, Saudi Arabia

## Abstract

**Objective:**

The study aimed to examine the factor structure and psychometric properties of ethical leadership questionnaire (ELQ) by using a healthcare professional sample in Saudi Arabia.

**Methods:**

A cross-sectional study was conducted, and a total of 387 healthcare professionals completed the 15-items ELQ questionnaire between 18 October, 2023 and 17 January, 2024. Exploratory factor analysis (EFA), confirmatory factor analysis (CFA), and a reliability test were performed on the obtained data.

**Results:**

It was found that the ELQ involved four factors including honesty (four items), integrity (four items), sets example (three items), and concern for values (four items). The study was able to explain 74.14% of the total variance. The model comparative fit index (CFI) was 0.952, the goodness-of-fit index (GFI) was 0.915, the standardized root mean squared residual (SRMR) was 0.0536, and the root mean square error of approximation (RMSEA) was 0.076. The reliability value was obtained by using Cronbach’s alpha, and the results of each subscale were higher than 0.80, which means they were deemed adequate and reliable. The results from these fit indices indicated that the four-factor model with EC offered the utmost fit.

**Conclusion:**

The ELQ is a reliable and valid instrument that can be used on this population to determine employees’ perceptions of their leaders’ ethical behavior.

## INTRODUCTION

Today, leadership is viewed as one of the most vital behavioral elements in any organization, and it allows the organization to pursue its objectives and contribute to workplace engagement.^1^ Leadership style refers to an individual’s behavioral qualities to inspire, instruct, and mentor subordinates to generate desired organizational outcomes.^2^ In addition, due to the inherent importance of their position and influence, leaders naturally serve as a source of moral guidance in organizational settings.^3^-^5^ When working with an ethical leader, healthcare professionals are exposed to a setting that values interpersonal justice, honesty, and sincere concern for others. As a result, employees experience more positive contentment and enjoyment in such situations, which helps them develop positive relationships and ethical work norms.^6^

Trevino et al. advocated that an ethical person and an ethical manager are closely associated with ethical leadership. An ethical person speaks to a leader’s characteristics in the workplace (e.g., fairness, honesty, and concern for others), whereas an ethical manager refers to managers using their positions and responsibilities to advance workplace morals.^7^ Ethical leaders create a work atmosphere that encourages social and professional engagement, increases employee satisfaction, and enhances job performance.^8^ Piccolo et al. recognized that ethical leaders support a culture where employees have more freedom.^9^ Hence, this circumstances incited interest in leadership theories of ethical concern.

Furthermore, due to the growing concern about ethical leadership in the workplace, assessing the ethical leadership behavior of healthcare professionals in Saudi Arabia is desirable. Over the years, little research has been conducted on ethical leadership in Saudi Arabia.^10^-^12^ In several countries, many researchers have used the ethical leadership questionnaire (ELQ) to assess the ethical behavior of employees.^13^-^15^ Several various facets of ethical leadership are described by this ELQ instrument, including integrity, fairness, setting an example, honesty, and concern for values.^16^ However, the factor structure and psychometric proprieties of the ELQ instrument have yet to be validated in Saudi Arabia. Therefore, the study aimed to examine factor structure and psychometric properties of the ELQ instrument. This research endeavor was undertaken to provide a validated and confirmed tool to assess followers’ opinions of leaders’ ethical behavior. Furthermore, this research may be helpful to most healthcare institutions in evaluating the ethical leadership behavior of leaders by their subordinates.

## METHODS

The study participants were junior-level healthcare professionals from public hospitals in Ha’il, Saudi Arabia. An anonymous questionnaire was employed between 18 October, 2023 and 17 January, 2024 to collect data from a sample of healthcare professional.

### Ethical considerations:

Before commencing this study, ethical approval was obtained from University of Ha’il Research Ethics Committee (Number: H-2020-196) and Ministry of Health (IRB Registration Number: H-08-L-074) in Saudi Arabia.

### Informed consent:

All participants provided their informed consent to participate in this study.

### Inclusion and exclusion criteria:

The study participants were those who had provided their informed consent. Study participants with less than one year of experience were excluded from this study.

### Study Instruments and Variables:

The ethical leadership questionnaire (ELQ) developed by Yukl et al.^17^ as an assessment tool was used (Table-I) to study various aspects of ethical leadership. This questionnaire includes 15 items with six anchors, strongly disagree (1) to strongly agree (6). A Cronbach’s alpha was calculated to assess the internal consistency among the 15 statements of the ELQ instrument. As a result, Cronbach’s alpha value for the ELQ scale was obtained to be 0.926, which showed that the scale was highly reliable.^18^ To determine the comprehensibility and understandability of the questionnaire, it was assessed by six working healthcare professionals in public hospitals in Hail city.

**Table-I:** Measured items of ethical leadership questionnaire (ELQ).

Item No.	Item Statements
ELQ1	My boss shows a strong concern for ethical and moral values.
ELQ2	My boss communicates clear ethical Standards for members.
ELQ3	My boss sets an example of ethical behavior in his/her decisions and actions.
ELQ4	My boss is honest and can be trusted to tell the truth.
ELQ5	My boss keeps his/her actions consistent with his/her stated values (“walks the talk”).
ELQ6	My boss is fair and unbiased when assigning tasks to members.
ELQ7	My boss can be trusted to carry out promises and commitments.
ELQ8	My boss insists on doing what is fair and ethical even when it is not easy.
ELQ9	My boss acknowledges mistakes and takes responsibility for them.
ELQ10	My boss regards honesty and integrity as important personal values.
ELQ11	My boss sets an example of dedication and self-sacrifice for the organization.
ELQ12	My boss opposes the use of unethical practices to increase performance.
ELQ13	My boss is fair and objective when evaluating member performance and providing rewards.
ELQ14	My boss puts the needs of others above his/her own self-interest.
ELQ15	My boss holds members accountable for using ethical practices in their work.

### Sampling Procedure and Data Collection:

The researchers used a convenience sampling method to collect the data. All junior healthcare managers in the selected public hospital were invited to participate in this survey. G*Power 3.1 software was used to determine the minimum needed sample size by using a 95% confidence level with a 5% error margin and a 0.20 effect size.^19^ This study required a minimum of 262 participants. Thus, the collected data were sufficient for this study because the data were collected from 387 junior healthcare managers working in public hospitals in Ha’il, Saudi Arabia.

### Data analysis:

The data analyses were performed by using SPSS 25.0 and AMOS 24.0 software. A principal component analysis with varimax rotation was used to perform an exploratory factor analysis (EFA) to extract the factors. Additionally, a confirmatory factor analysis (CFA) was conducted by using the maximum likelihood approach to evaluate the psychometric properties of the ELQ scale. In this process, single factor model, four factor model and four factors with EC model were developed and evaluated. The study used the relative chi-square (χ^2^/d.f.) value, goodness-of-fit index (GFI), comparative fit index (CFI), standardized root mean squared residual (SRMR), and root mean square error of approximation (RMSEA) to test a model’s goodness of fit.^20^ For the evidence of good psychometric properties factor loadings were used. A standardized correlation matrix was also used to ensure construct and discriminant validity of the instrument.^21^ Finally, the study utilized Cronbach’s alpha values to evaluate the general and individual factor reliability.^22^

## RESULTS

By applying a principal component analysis with the varimax-rotation method, an EFA was performed, and the results were presented in Table-II. Four factors were extracted from the resulting rotated factor matrix with an Eigenvalue ≥1, and an item statement had a factor loading of 0.5 or higher. The 15 items of the ELQ were grouped into four factors: honesty, integrity, sets example, and concern for values. The four factors together explained 74.14% of the total variance.

**Table-II:** Factor Structure of ethical leadership questionnaire (ELQ).

Factor	Items	Factor Loading
Honesty	ELQ1	0.821
	ELQ2	0.805
	ELQ4	0.694
	ELQ6	0.626
Integrity	ELQ5	0.729
	ELQ7	0.717
	ELQ8	0.847
	ELQ10	0.830
Sets example	ELQ3	0.827
	ELQ9	0.763
	ELQ11	0.804
Concern for values	ELQ12	0.591
	ELQ13	0.618
	ELQ14	0.0821
	ELQ15	0.743

**Fig.1:**
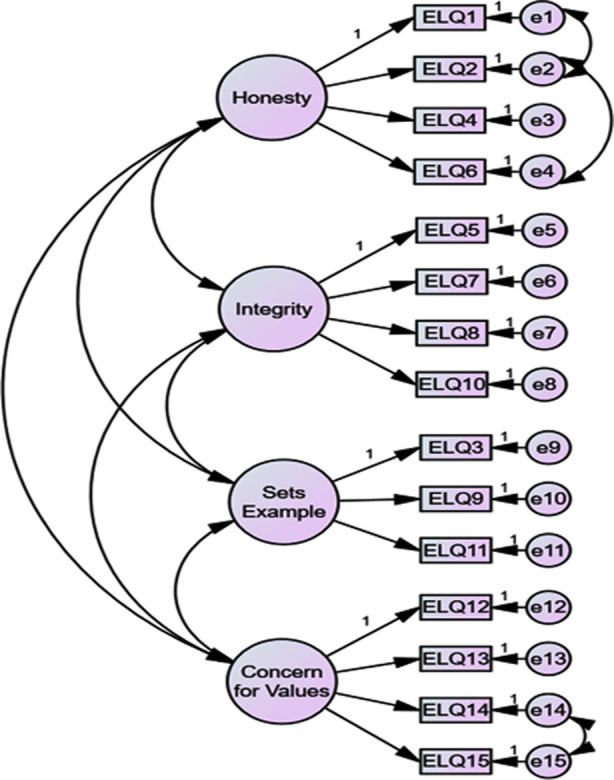
Factor model for the ethical leadership questionnaire (ELQ).

The CFA model’s goodness of fit indices are displayed in Table-III. The findings revealed that the unifactor model was not well fitted with the data; it caused the scale’s unidimensionality to be dismissed and advocated for the presence of differentiated measures for the other factors. In this model, the ratio between χ^2^ and the degrees of freedom (χ^2^/df) was 3.205. A ratio of <5 for the χ^2^/df statistic generally indicates that the observed data fit well within the proposed theoretical model, whereas a value <2 indicates a considerable modification. The model CFI was 0.952, and a CFI value of ≥0.95 was considered an excellent fit for the model. The GFI was 0.915, and a GFI value of ≥0.90 was considered an acceptable fit for the model. The standardized root mean squared residual (SRMR) was 0.0536, whereby values ≤0.05 indicate a good fit and values ≤10 indicate an acceptable fit. Finally, a root mean square error of approximation (RMSEA) of 0.76 was obtained, whereby RMSEA values ≤0.05 indicate a good fit and values ≤0.08 indicate an acceptable fit. According to these indices, the four-factor model with EC provided the best fit. Fig.1 depicted the final model structure with four EC factors.

**Table-III:** Goodness-of-fit indices for factor models.

Model	χ^2^/d.f.	CFI	GFI	SRMR	RMSEA (CI 90%)
Single factor	12.595	0.717	0.685	0.0994	0.173 (0.164–0.182)
Four factor	3.988	0.932	0.895	0.0566	0.088 (0.078–0.098)
Four factors with EC	3.205	0.952	0.915	0.0536	0.076 (0.065–0.086)

***Note:*** CFI = comparative fit index; GFI = goodness-of-fit index; SRMR = standardized root mean squared residual; RMSEA = root mean square error of approximation.

The standardized correlation coefficients between the factors and those between the variables and factors is presented in Table-IV. This measurement model’s factor loading was expected to exceed 0.60 because the ELQ scale was well established. The resulting factor loadings for each variable reached the required values. All the criteria were found to be statistically significant (p < 0.01). The lowest item loading was for ELQ 15, with a value of 0.625 in the subscale concern for values. The highest item loading, 0.878, was found for ELQ 13 in the subscale concern for values. Finally, the study findings supported the four factor EC model for the ELQ scale and presented a robust confirmation of the model’s construct validity.

**Table-IV:** Factor loadings for the four-factor model, including correlations between error terms.

Factor Items	Loading	R^2^
Honesty		
ELQ1	0.806	0.650
ELQ2	0.812	0.659
ELQ4	0.783	0.614
ELQ6	0.762	0.581
Integrity		
ELQ5	0.682	0.465
ELQ7	0.682	0.465
ELQ8	0.838	0.703
ELQ10	0.860	0.740
Sets example		
ELQ3	0.870	0.756
ELQ9	0.863	0.745
ELQ11	0.838	0.702
Concern for values		
ELQ12	0.835	0.696
ELQ13	0.878	0.772
ELQ14	0.679	0.461
ELQ15	0.625	0.390

***Note:*** R^2^ = percentage of variance explained.

Regarding the correlation analysis between the factors, moderate values (Table-V) were observed and ranged between 0.453 (correlation between concern for values and integrity subscales) and 0.838 (correlation between concern for values and honesty). The possibility that two elements represented the same dimension was ruled out by the absence of correlations ≥ 0.85. This result confirmed the instrument’s discriminant validity, as it indicated that the instrument had sufficiently diverse dimensions. Cronbach’s alpha value was obtained to measure the subscales’ reliability. The results in each subscale (Table-VI) were higher than 0.80, they were deemed adequate and reliable.

**Table-V:** Correlation matrix between factors.

	Honesty	Integrity	Sets example
Honesty			
Integrity	0.590		
Sets example	0.737	0.510	
Concern for values	0.838	0.453	0.704

**Table-VI:** Cronbach’s alpha values for subscales.

Factors	Number of items	Cronbach’s alpha
Honesty	4	0.874
Integrity	4	0.849
Sets example	3	0.892
Concern for values	4	0.853

## DISCUSSION

The results of the current study provided evidence of psychometric properties of the ELQ in Ha’il, Saudi Arabia, where studies on ethical leadership in healthcare are limited. The present study would support enhancing evaluation activities of ethical leadership in healthcare field in Saudi Arabia by evaluating the factor structure and psychometric aspects of the scale. Using a representative sample of healthcare professionals, the present study demonstrated the good psychometric properties of the ELQ scale.^17^ The internal reliability was also demonstrated to be good. The results presented quantitative evidence for the four stated factors: honesty, integrity, sets example, and concern for values.

Finally, present study findings support the theories and concepts of ethical leadership explained by many other authors. For example, Trevino et al. described ethical leadership’s components as honesty, fairness, concern for others, and ethics.^7^ Resick et al. empirically defined several elements of ethical leadership, including integrity, benevolence, motivation, encouragement, and empowerment.^23^ Wang and Hackett illustrated ethical leadership as virtue ethics, which includes intelligence, integrity, fairness, compassion, and honesty.^24^ Mitropoulou et al. stated that trust, honesty, humility, integrity, encouragement, altruism, sincerity, and uniformity with ethical behaviors are the dimensions of ethical leadership.^25^ Hence, the theory that describes ethical leadership was in accordance with the empirical results obtained from the subjects. Therefore, organizations can adopt this ELQ scale as a reliable instrument to measure employees’ perception of their leaders’ ethical behavior.

***Limitations:*** Present research was constrained to a limited geographical region with a smaller sample. A bigger sample size would allow for the presentation of more reliable results. Therefore, replicating this study in different geographical regions with a large sample size is important. Even measuring the concurrent validity of the ethical leadership questionnaire (ELQ) should be appropriate by correlating the ELQ with scales that measure the same concept.

## CONCLUSION

Ethical leadership questionnaire is found to be a reliable and valid scale to measure ethical leadership behavior in healthcare professionals in Saudi Arabia. Therefore, organizations can use this ELQ scale to evaluate employees’ perception of their leaders’ ethical behavior. Many studies have concluded that scale validity determination is an ongoing process. Besides addressing this limitation, further studies should be performed to measure the nomological validity of the ELQ scale to assess its association with followers’ psychological and behavioral outcomes. Ethical leadership’s consequences are diverse. Therefore, the predictive ability of ELQ scores should be confirmed in future studies. Finally, further research should be conducted to determine the effect of ethical leadership on multiple workplace issues such as satisfaction, commitment, turnover intention, performance, and productivity.
